# Understanding Emotions in Children with Developmental Disabilities during Robot Therapy Using EDA

**DOI:** 10.3390/s22145116

**Published:** 2022-07-07

**Authors:** Taisuke Nagae, Jaeryoung Lee

**Affiliations:** Department of Robotic Science and Technology, Chubu University, Kasugai 487-8501, Japan; tr20009-6774@sti.chubu.ac.jp

**Keywords:** HRI, social robot, emotions, children with autism, EDA, robot therapy

## Abstract

Recent technological advancements have led to the emergence of supportive robotics to help children with developmental disabilities become independent. In conventional research, in robot therapy, experiments are often conducted by operating the robot out of the subject’s sight. In this paper, robot therapy using a system that can autonomously recognize the emotions of a child with developmental disabilities and provide feedback was developed. The aim was to quantitatively infer emotional changes in children using skin conductance (EDA) during robot therapy. It was demonstrated that the robot could recognize emotions autonomously and provide feedback to the subjects. Additionally, a quantitative evaluation was conducted using EDA. By analyzing the symptoms related to developmental disorders, it may be possible to improve the recognition rate and tailor therapy based on symptoms.

## 1. Introduction

With the development of robot technology in recent years, research on educational support robots, which support or replace the education of teachers and parents, has been attracting substantial attention in educational facilities such as nursery schools and schools, as well as in homes. One in seven people worldwide have several disabilities. Among them, even though it varies depending on the definition, the portion of children was estimated as 5.1 percent of children aged 0–14 years-old have moderate or severe disabilities [1]. However, according to the World Health Organization, people with disabilities are particularly vulnerable to a lack of services such as healthcare, rehabilitation, support, and assistance [2]. Recent technological developments may offer the potential to alleviate these problems, meet user needs, and provide cheaper support systems. Therefore, assistive robotics that can help children with developmental disabilities to become independent have been introduced [3]. As support robotics to help children with developmental disabilities, robot therapy, in which robots and children with developmental disabilities interact with one another, has emerged. Robot therapy is effective for autistic children because they are not good at reading complicated facial expressions, but robots have a simpler structure than humans, so interpretation by autistic children enables easier active communication [4]. A study by Wood et al. showed the comparison of responses from children with a disability when they are in an interview with either robot or human [5]. Prior to this study, a similar experiment was conducted on healthy children, and it was found that the responses of children to the human and robot interviewers were very similar [6]. The results of the experiment with children with disabilities were same. It was demonstrated that they interacted with the human and robot interviewers in a very similar way. These results suggest that robot therapy may be effective for children with developmental disabilities.

### 1.1. Human–Robot Interaction and Robot Therapy

Human–robot interaction (HRI) is the field of understanding, designing, and evaluating robot systems that humans actually use or with which they coexist [7]. In recent years, robots have become more complex and sophisticated, and they have been integrated into everyday life, including workplaces, homes, hospitals, remote areas, dangerous environments, and battlefields [7,8,9]. As robots continue to enter people’s daily lives, it is of great importance to investigate the interactions between humans and robots. However, the structures, natures, and types of these interactions are dependent on the problems that are addressed in HRI research and development [7]. Moreover, in such interactions, robots communicate in the same manner as humans, using voice dialogue, gestures, and gazes. Thus, the term interaction in HRI is used in a fairly broad sense. In addition, effective analysis methods for HRI have not yet been elucidated or defined. Knowledge of human-to-human communication is often applied in this domain, and experiments may be designed or analyzed with reference to behavioral and clinical psychology [10].

In this context, the robot therapy field has been widely studied. Robot therapy is a psychotherapy technique mediated by robots that is aimed at improving autism and dementia. Regarding the therapeutic effect, research based on various verification methods has been conducted and quantitative knowledge is being accumulated [11]. Robot therapy is aimed at various people. In the field of elderly care, experiments have been conducted on the psychological and social effects of elderly people using lizard-type robots in nursing care facilities and, as a result, the mood of the elderly. This method was confirmed to be effective in improving depression [12]. Moreover, it takes elderly people’s preferences into account and has different appearances and functions [13].

### 1.2. Robots for Children with Autism

Autism spectrum disorder (ASD) is diagnosed when problems occur in daily life with the following symptoms. Individuals on the autism spectrum have difficulty in communication and speech delay, interpersonal interactions (communication with others), and obsession with particular objects [14]. ASD is a group of lifelong disorders that affect communication skills and the ability to understand social cues. It may also be associated with an intellectual disability or with other disorders or characteristics, such as hyperesthesia. Within this concept, the concept of ASD as a continuum has been redefined by including all of the various autism disorders and eliminating the boundaries with normality [15]. ASD is thought to be a congenital brain disorder. Other features include non-responsiveness to calls, hyperesthesia and bluntness, difficulty in sustaining concentration, poor attention and memory, difficulty in controlling emotions, immediate panic, and a dislike of environmental changes. Symptoms include those listed in [16].

Numerous studies in robots for children with autism and their findings present the effective and positive results of therapy by using the robots [17]. The reason to use the robots in this field is that, although it is difficult for autistic patients to read the complex facial expressions of humans, robots have a simpler structure than humans, which makes it easier for autistic children to interpret and communicate actively [4]. For example, Rudovic examined the effectiveness of robot therapy using three modalities (audio, visual, and autonomic physiology) [18]. Therefore, communication between a robot and an autistic child may help to improve the communication ability of the child. Besides this study, there are many studies and types of robots. We list the robots that have been used in robot therapy for children with autism, which is the focus of this study. Various designs have been implemented for robots aimed at patients with autism.

- KASPAR is a child-sized robot that was developed by the Adaptive Systems Group of the University of Hertfordshire, which can create human facial expressions and gestures. KASPAR has been recently updated to its fifth version for working more suitably in autism therapy [19]. With the fifth version of KASPAR, four children with autism participated in a game regarding emotions.
- Keepon is a robot with a yellow, round-shaped minimal body that was developed by the CareBots Project. The upper body has a head, left and right eyes, a wide-angle video camera equipped, and microphon-attached nose. With four degrees of freedom, Keepon expresses a state of mind with a gesture [20]. Twenty-five children participated in the environment, interacting with Keepon for 10 to 20 min, and the emotional communication was observed. Children made eye contact with, touched, and talked to Keepon [21].
- Muu is also famous as a simply designed robot that has only one eye in the robot body. It is covered with a soft material. It moves irregularly due to the internal spring, and is covered with a soft material. In the experiment with Muu [22], children with autism played with Muu for 5 to 10 min. This study was run once a month and observed for six months. The case study showed that Muu, described as an inanimate robot in the study, works for children with autism as a social agent.
- QTrobot, which was created by LuxAI, is an artificial-intelligence robot with an LCD face and a robot arm. It improves the emotion-recognition ability of autistic children by imitating their emotions with expressive faces and moving arms. The behavior of the robot is limited to a certain extent and the child is never denied. By using the attached tablet terminal, an individual can interact with QTrobot to increase the level of interest in the other party and to reduce confusion [23].
- CASTOR is a small humanoid that was produced by Diego et al. It offers a replicable robot platform that integrates the concepts of soft actuators and compliant mechanisms, and is aimed at real-world therapies, including physical interactions between children and robots [24].
- NAO is a small humanoid robot developed by Aldebaran Robotics (Softbank Robotics) that walks on two legs independently and has verbal and nonverbal communication functions [25]. This robot has been widely used and one of these fields is autism therapy, such as in study of Shamsuddin et al. [26]. They conducted a case study of autistic-child-and-robot interaction and compared his reactions in a normal class with people, by observation. The result shows that the child showed more active interactions when playing with robot then in the normal class.

The studies shows there are many different types of robots, but the results still indicate positive aspects for the children. These studies show that a robot could be an effective tool or agent for children with a developmental disability in their therapy session.

### 1.3. EDA in HRI

Electrodermal activity (EDA) is an electrical phenomenon in the skin [27] that has a psychological meaning. When the eccrine sweat glands are emotionally stimulated, they produce sweat, which has high electrical conductivity [28]. Therefore, the electrical properties of the skin change. As emotional stimuli cause changes in the skin conductance values through the activation of the eccrine sweat glands, EDA measurements can provide information for inferring emotions. Therefore, to quantify the degree of arousal caused by a stimulus, it is necessary to measure the amount of sweating, as indicated by an increase in the skin conductance value [29]. EDA measurements involve determining the electrical conductivity, resistance, impedance, and admittance of the skin [27]. When stimuli are recognized as important and cause an emotional response, the brain signals to the eccrine sweat glands through the sympathetic nerve branches of the autonomic nervous system, thereby activating sweating activity [30]. When sweat is secreted from the eccrine sweat glands, the skin conductance increases sharply and the skin conductance value increases. The process of increased skin conductance occurs within seconds of the stimulation occurring [31]. A sharp increase in skin conductance is known as the skin conductance response (SCR), electrocutaneous activity response, or peak. Previously, the SCR was commonly referred to as the galvanic skin reaction [32]. The arousal value is evaluated using Russell’s circumplex model, which defines emotions in a circle that is composed of comfort and discomfort, arousal, and drowsiness [33]. In this study, we use the emotions surprise, anger, joy, and sadness, indicated in red letters, for the four emotions with different degrees of arousal. In the experiment, the discrete wavelet transform and an SVM classifier were used for the EDA analysis. The reason for using the discrete wavelet transform is that the EDA signal is not periodic and its amplitude, phase, and frequency change. The wavelet transform is generally considered as effective in dealing with such unsteady signals [34]. The discrete wavelet transform is not easily affected by noise and it can easily be applied to unsteady signals [35]. The features that were used in this experiment are those that Rudovic demonstrated to be necessary for EDA analysis (z-normalized value, mean, SD, maximum, minimum, integral, slope, number of peaks, and number of zero intersections) [18]. The SVM is a supervised learning technique that can handle classification and regression, and it can perform the wavelet transform for EDA data. The SVM classifier was used to improve the emotion-classification performance significantly [36].

As mentioned in Section 1, children with autism are not good at reading complex facial expressions. However, by joining the robot with the therapist, it becomes possible to communicate more actively than when communicating only with the therapist, and emotional expression and communication abilities can be improved. In HRI, the robot is often operated by moving the robot to a location that is invisible to the subject (WoZ method [37]). The robot recognizes the emotion of the subject and feeds back the recognized emotion to the subject. Furthermore, in studies that have specialized in the developmental disorder known as autism, experiments produced quantified results using biometric data. However, in experiments that only included developmental disorders of children with developmental disabilities, a questionnaire was administered. Many experiments have been conducted in which guesses of emotional were made based on the above, and few have provided quantified results. Therefore, in this study, the interaction of the robot was autonomous, so that the emotions of the subject that were recognized could be fed back to the subject. Subsequently, the characteristics of the EDA in the emotions that were exhibited by a child with developmental disabilities during the interaction between the child and robot were extracted, and the emotions at that time were inferred from the EDA.

### 1.4. Contributions and Paper Overview

This work aims at developing a robot-assisted therapy system and analysis of emotions of children with autism during the therapy. A system was created in which a robot autonomously recognizes the emotions of a children with developmental disabilities and provides feedback to the therapist. During the therapy, the system understands children’s emotions by recognizing their faces. After that, we estimated their emotions by their EDA data. Besides a robot, various devices were needed for the precise measurement and performing of the therapy. By integrating various devices and a robot, the therapist was able to lead the robot-assisted therapy system. Moreover, the system was capable of providing feedback to the therapist so that he/she could understand the status of the children at the time. By analyzing their EDA, it was possible to quantitatively infer emotional changes during robot therapy. To the best of our knowledge, this is the first study to integrate all devices in the context of robot-assisted therapy for children with developmental disabilities. Previous work on robot-assisted therapy usually used an observation [26] as an assessment, and needed an operator for controlling the system [38]. In this work, by integrating all different devices into a system, it automatically recognizes and quantitatively assess the children’s emotional states. In addition, it became easier to use as the therapist was able to run the system. With this, we provide the future direction of robot-assisted-therapy research, that the therapist’s workload should be considered when developing the system and also the way to effectively use the analysis data of children’s emotional status during the therapy.

## 2. Methods

In previous studies, robot interactions were often manipulated by moving the robots out of sight of the subject (the WoZ method) [37]). Many studies used the WoZ method in robot-assisted autism therapy. In our previous study [39], we used WoZ method and the robot did not recognize the emotions of the subject and provide feedback. WoZ could help the robot interacts with human more naturally than interacting in an autonomous way. The therapy session, however, has a unique situation in comparison to general HRI, because of the presence of therapists. They are not the subjects of the experiment, but still the end user of the robot system and, at the same time, they are required to lead the sessions, instead of the robot operators. In this experiment, the robot recognized emotions autonomously and performed motions. The result of recognition was indicated by the light of the robot eyes so that the therapist understands if the robot correctly works. If the therapist judged that the recognition of robot is wrong or it is better to move on to other certain steps of session, they can control the robot directly through numeric keypad.

As a robot interacting with the children, we used NAO robot in this work. The NAO is a small humanoid capable of having a conversation with people and expressing emotions. This robot is used in therapy for the elderly and children with autism, and as a programming tool in many educational institutions and research facilities. In this experiment, the NAO moved only its upper body while sitting to express the four emotions.

**Figure 1:**
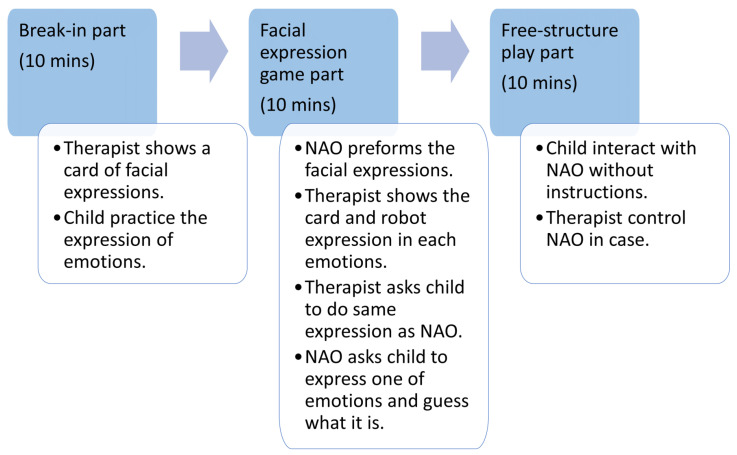
The procedure of the interaction between child and NAO robot.

**Figure 2:**
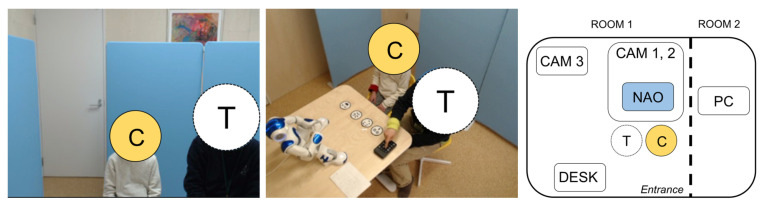
(**Left**) the scene in camera 1, C: child, T: therapist; (**center**) the scene in camera 3, C: child, T: therapist; (**right**) the experiment setup, C: child, T: therapist. At the very beginning of session, child and therapist sit in front of desk for around 10 min to relax before child was exposed to the robot. CAM 1 records only face and upper body of the participants. CAM 2 is used for recognizing the facial expressions. CAM 3 records the whole scene of session for reference.

Only the subject child and the therapist entered the room, and the NAO was enclosed in a partition to make it invisible at the start of the experiment. Each experiment was conducted for 10 min in three parts (the break-in, facial-expression game, and free parts,) as shown in Figure 1. A photograph of the experimental scene in the break-in and other parts obtained from camera 3 is presented in Figure 2. Both the facial-expression game and free parts faced the NAO.

- Break-in part: The therapist showed the child a facial expression card for each of the four emotions and the child practiced expressing the emotions.
- Facial-expression-game part: The NAO showed the child the facial expressions that were practiced in the break-in part. Thereafter, the therapist showed the facial-expression card that was identified by the NAO to the child and paired it with the facial expression performed by the NAO. The therapist then asked the child to perform the facial expressions specified by the NAO. The NAO instructed the child, Show me one of the four facial expressions, and the therapist encouraged the child to exhibit one. The NAO recognized the facial expressions and conveyed them to the subject. This was repeated until the correct answer was provided with four facial expressions.
- Free-structure-play part: When the facial-expression-game part was over, the therapist could freely operate the NAO using the numeric keypad. At this time, the NAO could indicate four facial expressions and compliment the subject.

Approximately 10 min were required in the break-in part to stabilize the biological signal data of the subject. The use of the facial-expression card only by the subject and therapist allowed the subject to recognize the facial expression that the card expressed. When the break-in part was over, the therapist was asked to move the partition and chair, and the child and therapist played the game with facial expressions facing the NAO. When the facial expression game was over, the therapist could move the NAO by operating the numeric keypad. Therefore, the subject moved the NAO freely and the robot played with the subject. The subjects of the experiment were 11 children with developmental disabilities (6 males, 5 females; mean age: 6.9 years; standard deviation: 1.1).

### 2.1. Facial Expression Game Part

**Figure 3:**
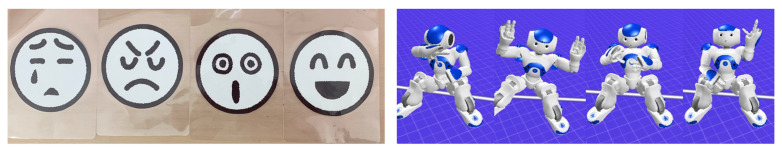
(**Left**) facial-expression cards, from left, sad, anger, surprise, happy; (**right**) the expressions by NAO, from left, sad, anger, surprise, happy.

The facial expression game is a method that is used for emotional expression education for autistic children with robotic support. In the facial expression game in this experiment, the therapist used cards with basic facial expressions (sad, anger, surprise and happy) as Figure 3 [40].

The NAO showed the expressions of these emotions to the child, and the therapist asked the child which emotions the robot expressed and made them aware of the emotions. Thereafter, the NAO asked the child to perform one of the four facial expressions (sadness, joy, anger, and surprise). The NAO read the child’s facial expression and told the child, “You have this facial expression out of the four facial expressions.” The NAO showed the emotion to the child, asked if it was correct, and asked the therapist to answer whether it was correct or incorrect using the numeric keypad provided to the therapist. If the answer was correct, the process of showing the facial expression was repeated using the three remaining facial expressions, and the process continued until the final facial expression was completed. If the recognized facial expression was different from the facial expression performed by the child, the four facial expressions were repeated. If the answers were correct, the facial expressions that were answered correctly were removed and the process continued until the final facial expression was reached. These steps were obtained from the theory-of-mind concept and designed to foster “sociological imagination”, which is a key challenge faced by many children [41]. The NAO movements were created for each of the four emotions using the “Choregraphe” for moving the NAO.

For “sad,” the NAO brings its arm in front of its face from the sitting position and makes a crying voice while making a human crying movement. For “joy,” the NAO shakes its hands and expresses joy in a loud voice. For “anger,” the NAO raises its arm and swings it back and forth while using a low voice. For “surprise,” the robot raises its hand slightly and lowers its face slightly behind, making a human hiccup-like voice.

### 2.2. Devices

We used multiple devices in the experiment, but all data were acquired from multiple laptops instead of one. Therefore, all E4, video, and audio data were recorded using coordinated universal time (UTC), making it easier to set the time with the other data for later analysis. The E4 wristband that was used to acquire the EDA in this experiment was connected to the E4 streaming server using a Bluetooth connection, and the acquired data were uploaded online to E4 Connect. Subsequently, the uploaded data were extracted in real time, and the UTC and Japan Standard Time (JST) were sequentially assigned to the acquired data in the text file. The network flow of the E4 wristband is described later. Furthermore, the NAO was connected to a personal computer using a wired cable. This was to prevent the connection with the NAO from being disrupted during the experiment.

In this experiment, an E4 wristband was used to obtain the EDA. The E4 wristband is a wristwatch-type terminal developed by Empatica that can acquire biological signals in real time. Sweating increases as the arousal value increases. The E4 wristband reads the amount of sweating from the skin potential and acquires it as the skin-potential activity. The unit of the acquired data was microseconds. In addition to the skin-potential activity, the blood volume pulses, acceleration, heart rate, and body temperature can also be measured. In this experiment, the EDA was used as the biological signal that could be obtained. It is also possible to measure the EDA in real time via a Bluetooth connection using a smartphone. However, this makes it difficult to match the time with other data such as video; thus, in the experiment, it was written to a text file using a program.

We used three web cameras (Logitech C922) in the experiment. The first was used to photograph the entire laboratory, the second captured the facial expressions of the subject, and the third captured the state and sound of the experiment from the front.

### 2.3. Integration of Systems

The robot therapy system included an app that controls the NAO, a mood mirror for facial recognition, a numeric keypad whereby the therapist operated the NOA, a recording app, and an app that acquires biological signals from the E4 wristband.

#### 2.3.1. NAO Interactive Application

The NAO app was created in Python using naoqi. First, the NAO introduced itself to the child and therapist. Thereafter, the NAO showed the child four facial expressions (sadness, joy, anger, and surprise). When the NAO was finished showing all of the emotions, the child was asked to show their facial expressions one by one. At that time, if the NAO could not recognize the facial expression and could not proceed, it was possible to proceed to the next expression by operating the numeric keypad. When the NAO had shown a series of facial expressions, the facial-expression game started. The NAO asked the child to express one of the facial expressions. At that time, the facial-expression data of the subject that was output from the mood mirror of the software for recognizing facial expressions was acquired, and the child was asked, “Is this the facial expression you are doing now?” Correct and incorrect answers were transmitted to the NAO by the therapist by operating the 10 keys. If the answer was correct, the game was played again with the three remaining facial expressions and if the answer was incorrect, the facial expression was repeated with the four facial expressions. Therefore, the game ended when the correct answer was provided four times (the NAO recognized all facial expressions). When the game was over, it was possible to operate the NAO with the numeric keypad when the child was told, “From here, play with the therapist’s teacher”. Thereafter, when the therapist pressed the end key, the program ended by saying “Thank you for playing with us today”.

#### 2.3.2. Recording Systems

The recording app was a Python app that was created using Tkinter, OpenCV, and other tools. When the app was initially run, the subject-information input screen appeared. When the subject information was entered and the OK button was clicked, the GUI for outputting the video was displayed, and the video was displayed in real time. If the camera was not connected, a dummy image was output. The video was not shot as a video, but the video that was captured by the camera using OpenCV was displayed as a video by superimposing it similar to a flip book. All captured images were saved in png format, and the image number and time when the image was captured (UTC and JST) were recorded in the log file. Audio data were recorded using Pyaudio. The times when the recording started and ended were recorded in JST in the log file.

#### 2.3.3. E4 Wristband System

The E4 app was also written in Python. A computer and E4 connected via Bluetooth were required in preparation for running the app. According to the specifications, when E4 was connected to the E4 streaming server, the acquisition of biological signals started, and the acquired biological-signal data were saved in E4 Connect via the Internet. In the E4 program, E4 Connect and telnet were connected, and the biological-signal data were constantly acquired and written to a text file separately for each piece of information. By visualizing the biological signals that were acquired at that time using a GUI, it was possible to confirm whether the data were acquired correctly at the same time. The acquired biological-signal data were also recorded as a set of acquired times (UTC and JST).

### 2.4. Analysis

Feng showed that the frequency analysis of raw EDA signals can provide a feature space for recognizing different emotions. To obtain a feature space for recognizing emotions, wavelet transform was performed on the center frequency and bandwidth parameters of the raw data to output the features, following which emotion classification was performed using an SVM classifier. It was found that the emotion classification performance was significantly improved by using the feature quantity after the wavelet transform and SVM classifier [36]. Ayata performed discrete wavelet transforms of EDA and brain waves, extracted the features, performed emotion recognition using machine learning, and compared machine-learning algorithms and feature-extraction methods [34]. Rudovic demonstrated the features (z-normalized value, mean, SD, maximum, minimum, integral, slope, number of peaks, and number of zero intersections) that are required for EDA analysis [18].

Therefore, in this experiment, the data that were obtained by extracting the features after performing the discrete wavelet transform and extracting the features as raw data of the EDA were subjected to machine learning. The reason for performing the wavelet transform is that biological signals are unsteady and change with time; therefore, the Fourier transform is inconvenient for analyzing EDA signals. EDA signals are nonperiodic and exhibit changes in the amplitude, phase, and frequency. Wavelet transforms are generally considered to be effective in dealing with such unsteady signals [34]. The discrete wavelet transform is a technique that was developed to overcome the shortcomings of the Fourier transform on unsteady signals, is less susceptible to noise, and can easily be applied to unsteady signals [35]. The machine learning we used in this work is a supervised learning technique using an SVM that can handle classification and regression, and it can deal with nonlinear data by margin maximization and the kernel method.

#### Features in EDA

The raw EDA data that were measured in this experiment and the data following the discrete wavelet transform were graphed. In this experiment, the data in the horizontal axis (time axis) direction were not constant because the time until the robot recognized the emotion of the subject varied. Noise data were removed from the data that were subjected to discrete wavelet transform and the number of peaks was reduced. The horizontal axis of the graph represents the time (s) and the vertical axis represents the measured EDA data [μS]. Moreover, the raw EDA data that were acquired in this experiment and the Z-values that were obtained from the data following the wavelet transform were graphed. This graph indicates the time on the horizontal axis (s) and Z-values on the vertical axis.

### 2.5. Hypothesis

As mentioned, Feng extracted the features following wavelet transform and demonstrated that the classification performance of the emotions was significantly improved by using the SVM classifier. Subsequently, Ayata performed discrete wavelet transform of the EDA and brain waves, extracted the features, and performed emotion recognition using machine learning to show the effectiveness of the discrete wavelet transform. Therefore, in these experimental data, it was considered that the emotion recognition is higher in the data that were obtained by performing wavelet transform and then extracting the features than in the method of extracting the features from the raw data.

## 3. Results

**Figure 4:**
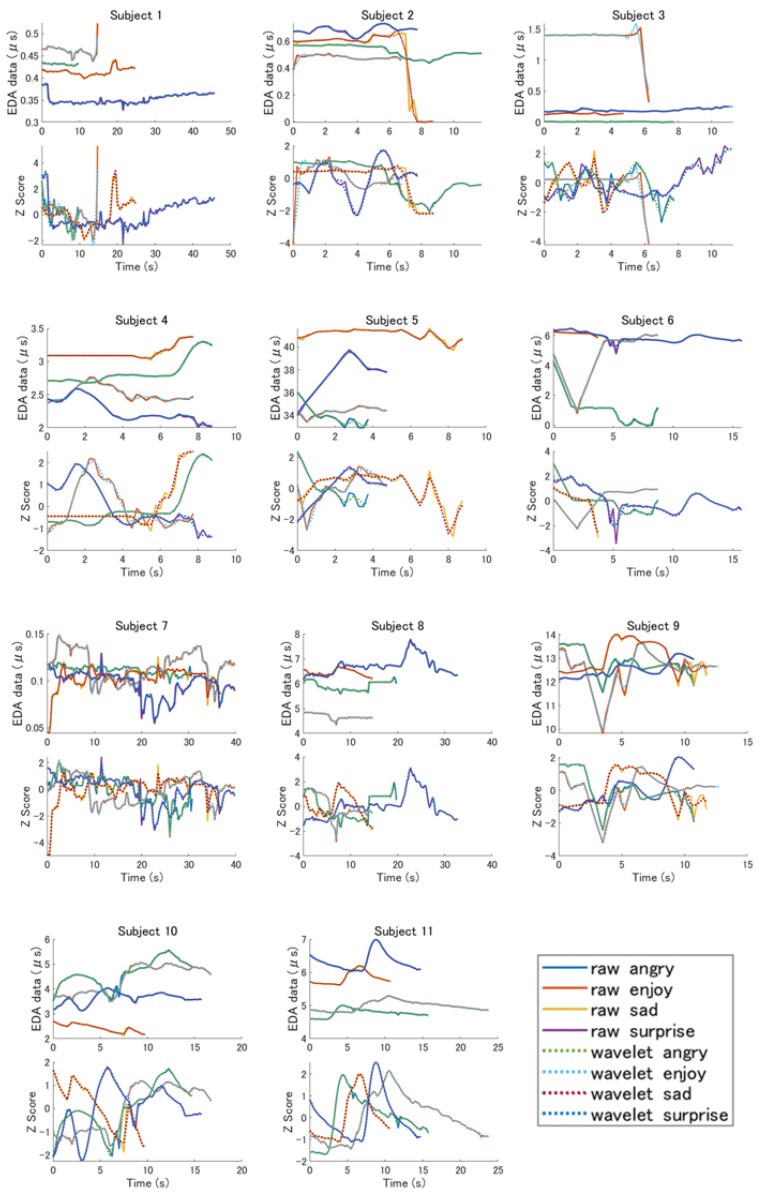
Raw and wavelet transferred data, and z scores of all subjects.

The EDA data and Z-values of the subjects are presented in Figure 4. The mean, standard deviation, maximum, minimum, integral, slope, and zero intersection that were obtained from the EDA data are listed in Table 1 and Table 2. The top-four datasets in the tables are the features that were calculated from the raw data, whereas the bottom four are the features that were obtained following the wavelet transform.

### 3.1. Features in Each Subject

- When the average values of the raw data of subject 1 and the EDA data following wavelet transform were arranged in descending order, both were in the order of “joy,” “anger,” “sadness,” and “surprise.” As a result, in Russell’s circumplex model, “surprise,” with the highest arousal value, was the lowest.
- When the average values of the EDA data from the raw data of subject 2 were arranged in descending order, the order was “surprise,” “anger,” “sadness,” and “joy.” However, when the EDA data following wavelet transform were sorted, the order was “surprise,” “sadness,” “joy,” and “anger.” In both of these results, the arousal values of Russell’s circumplex model were not in descending order, but the changes appeared in the order of the mean value of the EDA data before and after the wavelet transform was applied.
- When the average values of the raw data of subject 3 and the EDA data following wavelet transform were arranged in descending order, both were in the order of “joy,” “surprise,” “sadness,” and “anger.” This result was not in descending order of the arousal value in Russell’s model.
- When the average values of the raw data of subject 4 and the EDA data following wavelet transform were arranged in descending order, both were in the order of “sadness,” “anger,” “joy,” and “surprise.” As a result, the lowest on the arousal axis of Russell’s model, “sad,” was the highest, and the highest, “surprise,” was the lowest, on the arousal axis.
- The EDA data of subject 5 had a higher EDA value for all data than the other subjects. When the average values of the raw data and the EDA data following wavelet transform were arranged in descending order, they were both in the order of “sad,” “surprise,” “joy,” and “anger.” In this result, “sad,” which should have the lowest arousal value in Russell’s model, was the highest.
- When the average values of the raw data of subject 6 and the EDA data following wavelet transform were arranged in descending order, they were both in the order of “surprise,” “sadness,” “joy,” and “anger.” This result demonstrates that “sad,” which had the lowest arousal value in Russell’s model, was second highest.
- The EDA data of subject 7 did not visually differ for each emotion. When the average values of the raw data and the EDA data following wavelet transform were arranged in descending order, they were both in the order of “joy,” “anger,” “sadness,” and “surprise.” This result indicates that “surprise,” which had the highest arousal value, was the highest in Russell’s model.
- When the average values of the raw data of subject 8 and the EDA data following wavelet transform were arranged in descending order, both were in the order of “surprise,” “sadness,” “anger,” and “joy.” As a result, “sadness,” which was the lowest arousal value in Russell’s model, was the second highest.
- When the average values of the raw data of subject 9 and the EDA data following wavelet transform were arranged in descending order, both were in the order of “sadness,” “anger,” “surprise,” and “joy.” This result was the highest for “sadness,” which had the lowest arousal value in Russell’s model.
- When the average values of the raw data of subject 10 and the EDA data following wavelet transform were arranged in descending order, both were in the order of “anger,” “joy,” “surprise,” and “sadness.” According to this result, “sadness,” which had the lowest arousal value in Russell’s model, was the only one with the lowest arousal value.
- When the average values of the raw data of subject 11 and the EDA data following wavelet transform were arranged in descending order, both were in the order of “surprise,” “sadness,” “joy,” and “anger.” This result was not in descending order of the arousal value in Russell’s model. Moreover, in this experiment, the time that was required to acquire the emotions was not constant, but subject 11 exhibited a significant change for each emotion according to the Z-value.

### 3.2. Average of EDA Data

**Figure 5:**
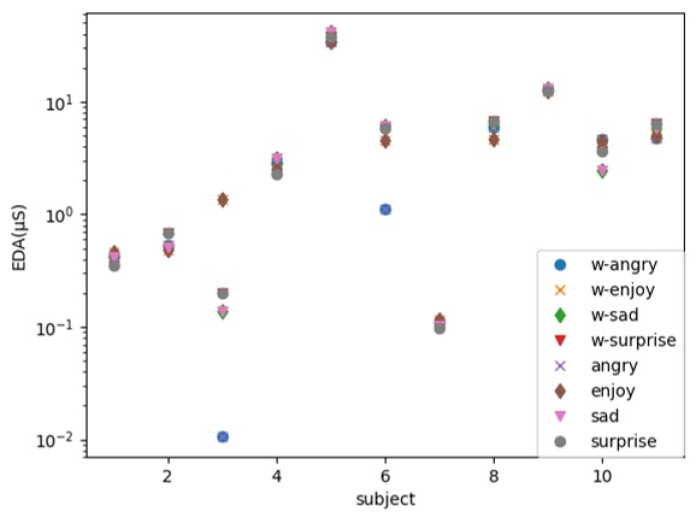
Average of recognition rate in all subjects.

The average values of the data that were acquired in this experiment for each emotion are depicted in Figure 5. One of the subjects had a larger value than the other subjects, we output the data in a log–log graph so that the distribution could be understood. When the EDA was sorted in descending order according the mean value of the raw data and the mean value of the data after the wavelet transform was performed, the raw data and wavelet transform data were the same, except for those of one person. In Russell’s circumplex model, when the emotional arousal values were arranged in descending order, the order was “surprise,” “anger,” “joy,” and “sadness.” None of the EDA data were arranged in the order of the arousal value.

### 3.3. Recognition Rate

**Figure 6:**
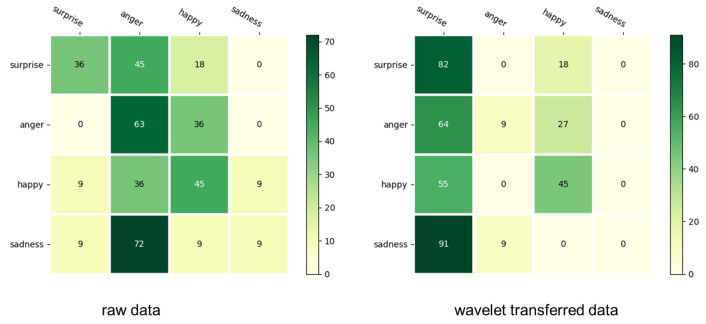
(**Left**) recognition rate for each emotion in raw data; (**right**) recognition rate for each emotion in wavelet-transferred data.

The results of the machine learning (SVM) using the data that were obtained by extracting the features from the raw data indicate that the emotion recognition rate was 38.6%. The confusion matrix is presented in Table 3 and the recognition rate for each emotion is shown in Figure 6. The results of performing the wavelet transform, extracting the features, and performing machine learning indicate that the emotion recognition rate was 34.0%. The confusion matrix is presented in Table 3 and the recognition rate for each emotion is shown in Figure 4. In the confusion matrix, the emotion that was identified appears in the horizontal direction and the actual emotion is shown in the vertical direction, and the heat map is rounded to the nearest number. As illustrated in the left of Figure 6, for the analysis with the raw data, the recognition rate of “anger” was higher than that of the other emotions. As can be observed from the right of Figure 6, when the feature quantity was extracted following wavelet transform and emotion identification was performed, the identification rate of “surprise” was approximately 82%, which was higher than that of the other emotions. However, the emotional recognition rate of “sad” was less than 10% in both cases.

## 4. Discussion

In this experiment, a robot autonomously recognized emotions and could feed the recognized emotions back to the subject using the color of its eyes. However, the emotion recognition rate was extremely low. This is because the number of subjects was small and the data of various children with developmental disabilities were collectively identified without distinction. Furthermore, according to the emotion recognition results, the emotion recognition of “sadness” was less than 10% in both cases. “Sadness” had the lowest arousal value among the emotions used in Russell’s circumplex model. However, “surprise” had the highest arousal value among the emotions used. The “sad” emotions that were identified from Figure 2 and Figure 3 were classified as “anger” in the raw data and “surprise” after the discrete wavelet transform. When the features were extracted from the data were subjected to discrete wavelet transform and identified, over 50% of all four emotions were recognized as “surprise.” This may be related to the fact that children with developmental disabilities are not good at expressing their emotions. According to the experimental results, when the mean values of the EDA data for each of the four emotions were sorted in descending order, none of the data were arranged in descending order of the arousal value in Russell’s model. In Russell’s circumplex model, “sad,” with the lowest arousal value, often appeared in the top two when the average values of the EDA data were arranged in descending order; thus, the subjects expressed “sad” emotions. This could be because subjects could not express this emotion well, even if they believed they were doing so.

## 5. Conclusions

According to the experimental results, it was possible for the robot to recognize emotions autonomously and to feed the recognized emotions back to the subjects. However, emotion recognition using the EDA data exhibited a low recognition rate and posed a problem. In this experiment, the number of subjects was small; therefore, it was not possible to distinguish between the symptoms of children with developmental disorders. Furthermore, “sad” was classified as the emotion with the lowest arousal value among the emotions used in Russell’s circumplex model, but it was the emotion with the highest arousal value in this analysis result. Based on these results, it is necessary to analyze the symptoms of children with developmental disabilities.

Differences exist in the strengths and weaknesses of emotional expressions depending on the symptoms of children with developmental disorders. As the number of subjects in this experiment was small, the analysis was performed not by symptom-based analysis of children with developmental disabilities, but according to the group of children with developmental disabilities. Therefore, the recognition rate of emotions was considered to be low. Following the study of Robins [42], the types of development disabilities affect interactions with a robot, which takes the symptom-based analysis into account. In future experiments, by increasing the number of subjects and analyzing the symptoms of children with developmental disabilities, the characteristics of each symptom will appear in the EDA data, and it will be possible to identify emotions with a higher recognition rate than that obtained in this experiment. Moreover, in this experiment, the recognition rate was higher when the raw data were analyzed without the discrete wavelet transform; however, this also increased with the number of subjects. Thus, it is necessary to investigate whether the recognition rate can be improved by symptom analysis.

## Figures and Tables

**Table 1:** Features in EDA data of all subjects (1–8).

S1	Emotion	Mean	SD	Max.	Min.	Integral	Slope	Peak	Zero c.
Raw	Anger	0.43149	0.00203	0.43654	0.42758	4.20634	−0.00050	7	0
	Joy	0.46136	0.01170	0.52360	0.43782	6.79693	−0.00096	10	0
	Sadness	0.41464	0.00817	0.44039	0.39814	10.2608	0.00036	17	0
	Surprise	0.35291	0.01088	0.38790	0.32773	16.1393	0.00035	42	0
Wavelet	Anger	0.43150	0.00197	0.43669	0.42761	4.20636	−0.0005	6	0
	Joy	0.46117	0.00949	0.49402	0.43901	6.79779	−0.00103	8	0
	Sadness	0.41465	0.00807	0.44204	0.39984	10.2610	0.00036	12	0
	Surprise	0.35291	0.01075	0.38882	0.33504	16.1395	0.00034	20	0
**S2**	**Emotion**	**Mean**	**SD**	**Max.**	**Min.**	**Integral**	**Slope**	**Peak**	**Zero c.**
Raw	Anger	0.52904	0.04389	0.57608	0.4391	6.21269	−0.00947	7	0
	Joy	0.47970	0.02236	0.50951	0.38790	3.25197	−0.00155	6	0
	Sadness	0.50582	0.23713	0.66058	0	4.47646	−0.05765	6	0
	Surprise	0.68445	0.02883	0.73381	0.61860	5.30536	0.00308	5	0
Wavelet	Anger	0.43150	0.00197	0.43669	0.42761	4.20636	−0.00050	6	0
	Joy	0.47925	0.02133	0.50300	0.40257	3.24552	−0.00093	4	0
	Sadness	0.50591	0.23201	0.68548	−0.00423	4.47763	−0.0577	6	2
	Surprise	0.68445	0.02851	0.73536	0.61731	5.30490	0.00314	4	0
Raw	Anger	0.01057	0.00342	0.01537	0.00129	0.08179	−0.00065	8	0
	Joy	1.34860	−0.05213	1.52668	0.32300	8.54974	−0.05213	6	0
	Sadness	0.13925	0.01050	0.16262	0.12036	0.66230	−0.00079	4	0
	Surprise	0.19759	0.02413	0.25865	0.16774	2.16917	0.00464	12	0
Wavelet	Anger	0.01058	0.00307	0.01495	0.00315	0.08181	−0.00063	3	0
	Joy	1.35171	0.21511	1.59836	0.50761	8.54695	−0.04914	5	0
	Sadness	0.13916	0.00998	0.15575	0.11871	0.66207	−0.00076	2	0
	Surprise	0.19887	0.02437	0.25580	0.16911	2.23338	0.00496	6	0
**S4**	**Emotion**	**Mean**	**SD**	**Max.**	**Min.**	**Integral**	**Slope**	**Peak**	**Zero c.**
Raw	Anger	2.84252	0.19124	3.30177	2.67971	24.8388	0.05799	6	0
	Joy	2.51112	0.11441	2.7619	2.36640	19.4837	−0.01499	5	0
	Sadness	3.13211	0.09746	3.37641	3.02315	24.2489	0.02746	5	0
	Surprise	2.25934	0.16982	2.59158	2.02948	19.7752	−0.05478	4	0
Wavelet	Anger	2.84270	0.19162	3.29200	2.67752	24.8380	0.05815	5	0
	Joy	2.51067	0.11261	2.74637	2.37213	19.48200	−0.01502	3	0
	Sadness	3.13210	0.09633	3.37472	3.05084	24.2487	0.02746	2	0
	Surprise	2.25934	0.16982	2.59158	2.02948	19.7752	−0.05478	4	0
**S5**	**Emotion**	**Mean**	**SD**	**Max.**	**Min.**	**Integral**	**Slope**	**Peak**	**Zero c.**
Raw	Anger	33.9771	0.88651	36.0453	32.9277	127.196	−0.62724	2	0
	Joy	34.3520	0.34325	34.8394	33.4283	163.152	0.16487	4	0
	Sadness	41.1273	0.44909	41.6308	39.7299	359.940	−0.08252	6	0
	Surprise	37.5190	1.59681	39.7418	34.0201	178.622	0.77806	2	0
Wavelet	Anger	33.9707	0.87512	36.0630	33.0540	127.000	−0.64781	2	0
	Joy	34.3571	0.33700	34.7807	33.5023	163.000	0.15909	2	0
	Sadness	41.1272	0.43593	41.5292	39.8615	360.000	−0.08425	5	0
	Surprise	37.5162	1.60449	39.5258	34.0479	179.000	0.78431	1	0
**S6**	**Emotion**	**Mean**	**SD**	**Max.**	**Min.**	**Integral**	**Slope**	**Peak**	**Zero c.**
Raw	Anger	1.11767	1.05655	4.30188	0	9.37177	−0.32224	5	0
	Joy	4.50357	1.62758	6.06718	0.80439	39.1786	0.47677	3	0
	Sadness	6.14001	0.10694	6.24970	5.82469	23.0507	−0.07339	0	0
	Surprise	7.87500	0.30894	6.47427	4.79894	92.4318	−0.03553	9	0
Wavelet	Anger	1.11760	1.05880	4.29840	−0.16029	9.38515	−0.32420	4	4
	Joy	4.50490	1.62674	6.03391	0.96924	39.1920	0.47578	3	0
	Sadness	6.14143	0.10092	6.24910	5.87374	23.0504	−0.07152	1	0
	Surprise	5.87119	0.29414	6.47482	5.25587	92.4337	−0.03549	6	0
**S7**	**Emotion**	**Mean**	**SD**	**Max.**	**Min.**	**Integral**	**Slope**	**Peak**	**Zero c.**
Raw	Anger	0.11154	0.00396	0.11904	0.09728	3.42962	−0.00034	23	0
	Joy	0.11540	0.01626	0.14848	0.07808	4.58717	−0.00020	27	0
	Sadness	0.10286	0.01197	0.12544	0.04480	3.78928	0.00033	29	0
	Surprise	0.09654	0.01340	0.12928	0.05504	3.83536	−0.00054	29	0
Wavelet	Anger	0.11153	0.00383	0.11988	0.09826	3.42974	−0.00034	18	0
	Joy	0.11540	0.01612	0.14951	0.08161	4.58725	−0.00020	22	0
	Sadness	0.10286	0.01176	0.11850	0.04423	3.78926	0.00033	25	0
	Surprise	0.09654	0.01310	0.12113	0.05889	3.83521	−0.00054	22	0
**S8**	**Emotion**	**Mean**	**SD**	**Max.**	**Min.**	**Integral**	**Slope**	**Peak**	**Zero c.**
Raw	Anger	5.91103	0.17873	6.25988	5.60335	116.714	0.00314	9	0
	Joy	4.68259	0.11362	4.84751	4.35351	69.0545	−0.01744	12	0
	Sadness	6.43958	0.12888	6.68935	6.19407	94.9965	−0.00588	7	0
	Surprise	6.70039	0.34390	7.77854	6.17104	219.547	0.01438	21	0
Wavelet	Anger	5.91138	0.17695	6.19766	5.61145	117.000	0.00329	12	0
	Joy	4.68262	0.11302	4.84765	4.39869	69.1000	−0.01745	9	0
	Sadness	6.43941	0.12853	6.68754	6.21155	95.0000	−0.00590	6	0
	Surprise	6.70031	0.34300	7.69505	6.15898	220.000	0.01438	18	0

**Table 2:** Features in EDA data of all subjects (9–11).

S9	Emotion	Mean	SD	Max.	Min.	Integral	Slope	Peak	Zero c.
Raw	Anger	12.8038	0.50481	13.6106	11.5710	137.666	−0.09282	8	0
	Joy	12.4684	0.82407	13.6375	9.82250	155.723	0.04463	7	0
	Sadness	13.0196	0.65215	14.0324	11.8252	153.147	0.01768	9	0
	Surprise	12.5225	0.33536	13.2030	12.0830	134.619	0.08715	7	0
Wavelet	Anger	12.8047	0.49672	13.5953	11.6240	138.000	−0.09210	6	0
	Joy	12.4711	0.80853	13.6875	9.95330	159.000	0.04415	5	0
	Sadness	13.0218	0.63702	14.0203	11.9910	153.000	0.01878	6	0
	Surprise	12.5225	0.33543	13.2109	12.0873	135.000	0.08741	5	0
**S10**	**Emotion**	**Mean**	**SD**	**Max.**	**Min.**	**Integral**	**Slope**	**Peak**	**Zero c.**
Raw	Anger	4.62988	0.54054	5.56541	3.50872	68.3935	0.09091	5	0
	Joy	4.39484	0.55553	5.06021	3.48950	73.6648	0.09339	12	0
	Sadness	2.42979	0.16078	2.69310	2.12594	23.6907	−0.04779	3	0
	Surprise	3.63377	0.22659	4.04169	3.12761	57.2970	0.01891	7	0
Wavelet	Anger	4.62950	0.53832	5.51474	3.49822	68.3901	0.09107	4	0
	Joy	4.39519	0.55496	5.05378	3.51319	73.6676	0.09337	7	0
	Sadness	2.42985	0.15934	2.69442	2.16082	23.6916	−0.04788	2	0
	Surprise	3.63373	0.22605	4.02052	3.11828	57.2966	0.01894	4	0
**S11**	**Emotion**	**Mean**	**SD**	**Max.**	**Min.**	**Integral**	**Slope**	**Peak**	**Zero c.**
Raw data	Anger	4.77608	0.11609	5.00440	4.58069	75.2565	0.00348	8	0
	Joy	4.98669	0.14246	5.29370	4.78678	118.464	0.00435	13	0
	Sadness	5.82886	0.18590	6.20250	5.62175	62.6857	0.02628	5	0
	Surprise	6.32382	0.26272	6.99116	6.03171	93.2778	0.00233	4	0
Wavelet	Anger	4.77612	0.11578	5.00377	4.58275	75.2560	0.00350	7	0
	Joy	4.98670	0.14240	5.29604	4.78298	118.000	0.00435	9	0
	Sadness	5.82887	0.18581	6.19785	5.62209	62.6856	0.02627	2	0
	Surprise	6.32389	0.26278	6.98930	6.03438	93.2793	0.00228	3	0

**Table 3:** Confusion matrix of raw data and wavelet data.

Confusion Matrix	Emotion	Surprise	Anger	Happy	Sad
Raw data	surprise	4	5	2	0
	anger	0	7	4	0
	happy	1	4	5	1
	sad	1	8	1	1
Wavelet	surprise	9	0	2	0
	anger	7	1	3	0
	happy	6	0	5	0
	sad	10	1	0	0
